# Evaluation of Paediatric Critical Care Needs and Practice in Nigeria: Paediatric Residents' Perspective

**DOI:** 10.1155/2021/2000140

**Published:** 2021-08-31

**Authors:** Moses Temidayo Abiodun, Fidelis E. Eki-Udoko

**Affiliations:** Paediatric Emergency & Critical Care Division, Department of Child Health, University of Benin Teaching Hospital, & School of Medicine, University of Benin, Benin City, Nigeria

## Abstract

**Background:**

There is a dire need for paediatric critical care (PCC) services, but their availability in tertiary hospitals in Nigeria is not well defined.

**Objective:**

We evaluated self-reported PCC practice, resources, and perceived challenges in various zones of the country, using paediatric residents' perspective.

**Methods:**

This is a descriptive cross-sectional survey, carried out at an Intensive Course in Paediatrics at the University of Benin Teaching Hospital, Nigeria. Participants' PCC practice and perceived adequacy of PCC resources and services were assessed using a 100 mm uncalibrated visual analogue scale (VAS). A comparison between northern and southern zones was done. A 2-sided *p* value < 0.05 was considered significant.

**Results:**

A total of 143 residents participated in the study, 37.1% of them were male, and 62.9% were female. Their mean age was 34.6 ± 3.2 years. They were mainly (86.7%) from federal institutions across the country. Less than a half (46.7%) of the trainees attended to critically ill children daily, but only 4 out of every 10 respondents stated that such severely ill children survived till hospital discharge; 12.1% of the trainees had PICUs in their institutions. Financial constraints hindered PICU admissions. PCC staff were relatively fewer in northern zones than southern zones (*p* < 0.05). Their perceived adequacy of PCC equipment and services were low (VAS scores 32.7 ± 2.6 and 30.9 ± 2.8, respectively) with a strong positive correlation between the two measurements (*r* *=* 0.839; *p* < 0.001).

**Conclusion:**

There is an unmet need for PCC practice in Nigerian tertiary hospitals with a resultant low survival rate of critically ill children. PCC training curricula and improved critical care resources are desirable in the setting.

## 1. Introduction

Paediatric critical care (PCC) or intensive care is a relatively new medical specialty, especially in low- and middle-income countries [1]. PCC ensures optimal monitoring and organ-system support of critically ill children while taking cognizance of their unique anatomic and developmental stages. Recovery from childhood critical illnesses is dependent on clinical-laboratory monitoring, prompt escalation of clinical care, effective team dynamics, and multidisciplinary care as well as adequate communication with caregivers [1, 2]. However, PCC is not readily available in a resource-limited setting, despite the dire need for such levels of care [2, 3]. A multicountry survey by Muttalib et al. PALISI Global Health Subgroup reported major gaps in the availability of essential PCC resources in resource-limited settings [4]. A national audit of critical care resources in South Africa by Bhagwanjee and Scribante [5] found that, regarding the public sector ICU bed, the population ratio ranged from less than 1 : 20,000 to 1 : 80,000 but less than one-fifth of beds are dedicated to paediatric and neonatal patients. Also, Touray et al. [6] in Gambia reported in 2018 that only one hospital among seven surveyed public hospitals (treating more than 50 critically ill patients a month) had a dedicated intensive care unit, resulting in an estimated 0.4 ICU beds/100,000 population in the country, while Siaw-Frimpong et al. [7] in Ghana reported 0.5 ICU beds/100,000 people in 2020. Again, Atumanya et al. [8] in Uganda reported 1.3 ICU beds per million population in a recent survey, showing limited accessibility to critical care services in the country. Likewise, a recent single-centre study in southern Nigeria showed that 33.3% of children admitted into the Emergency Unit required critical care but only 2.8% of these critically ill children were transferred to the hospital's general ICU due to limited relevant resources and financial constraints [3].

Paucity of critical care services worsens the outcome of childhood illnesses in low- and middle-income countries. In a review of PCC in resource-limited settings, Turner et al. [1] affirmed that an upgrade of critical care resources and services is needed to reduce child mortality in the region. A comparative study of two PICUs by Abdelatif et al. [9] showed that the inadequate structure and higher burden of severely ill children at a resource-limited Egyptian PICU contributed to higher mortality in it than in a resource-rich Japanese PICU. Also, considering the multilevel, family-societal factors that promote late presentation of sick children to health facilities in medically underserved regions, prompt access to optimal critical care remains an important strategy to reduce childhood mortality in the setting [10, 11]. In a 5-year review of hospitalized children in an ICU in Cameroon, Nguefack et al. [12] reported that deaths occurred mainly (90.0%) within 72 hours of admission and malnutrition was the underlying cause of death in nearly a quarter of the patients. Likewise, Akindolire and Tongo [3] in Ibadan southern Nigeria reported that almost a quarter of their patients died within 48 hours of presentation in the Emergency Unit, despite the majority of their illnesses being potentially reversible with prompt critical care [3]. This highlights the fact that children often present in extreme condition in the setting, requiring specialized multisystemic supports to survive the illness. Assessment of available critical care resources in the health system is therefore a major step towards improving paediatric health indices in the subregion [1, 9].

Considering the foregoing, this national survey evaluated PCC practice and resources in Nigeria, using frontline clinician's viewpoints. We hypothesized that PCC services are inadequate in all geopolitical zones of the country. We aimed to determine the burden (frequency/types/outcome) of critical illnesses seen by paediatric residents in the preceding 3 weeks as well as PCC resources available for their management. Hence, this study quantified the existing gap in critical care in the Nigerian health system and harvested relevant recommendations.

## 2. Methods

### 2.1. Study Setting and Participants

The study took place from March 11 to March 22, 2019, at the Oba Akenzua Complex multipurpose hall of the University of Benin Teaching Hospital (UBTH), Benin City, Nigeria. The participants were paediatric resident doctors from training institutions in different zones of the country attending the Intensive Course in Paediatrics of the National Postgraduate Medical College of Nigeria (NPMCN) at the venue. Respondents' institutions were classified into federal, state, and private, in the various geopolitical zones in the country.

### 2.2. Study Design and Sample Size

This was a descriptive cross-sectional study. It was a total population study of all consenting resident doctors attending the NPMCN's Intensive Course in Paediatrics.

### 2.3. Data Collection

The questionnaire was designed based on the principal investigator's conception of the study and relevant literature searches [3, 13]. Each item on the questionnaire was reviewed independently by the researchers to ensure clarity. It comprised sections on (a) sociodemographic and practice characteristics, (b) burden of critical illnesses treated in the preceding 3 weeks, (c) availability of physical/human resources for PCC at the training institutions, and (d) residents' perceived adequacy of critical care practice using 100* *mm uncalibrated visual analogue scales (VAS). Critical illnesses were classified based on the Paediatric Assessment Triangle (PAT) categories [13]. Perceived challenges and recommendations to improve critical care practice in the setting were elicited using open-ended questions. The critical illness description subscale was a six-item Likert scale in which items (e.g., “They are mainly infectious” and “Late presentation is common”) were answered on a four-point scale, from “strongly agree” to “strongly disagree.” Information about the study was provided to the paediatric trainees at the conference registration desk, emphasizing voluntary participation. The pretested self-administered questionnaire with a study information statement was included in their conference package. Also, they were centrally reminded during lunchtime daily in the first week of the conference, and a research assistant went around the hall to retrieve filled questionnaires. The response rate was 100%.

### 2.4. Pretesting

The initial survey instrument was pretested on 15 paediatric residents at the Department of Child Health, UBTH, Benin City, to ensure clarity of its items and validity. On feedback, all the questionnaire items were readily understood by the trainees. The computed reliability rating (*Cronbach's alpha*) of the critical illness description subscale was adequate (0.68). No further changes were made to the questionnaire.

### 2.5. Statistical Analysis

The data were analysed using the Software Package for Social Science (SPSS) version 20.0 (Windows Inc., Chicago, IL, USA). Categorized data were presented on frequency tables. Percentages are denominated on the total number of respondents per variable. A mean score of 2.5 on the Likert scales was taken as adequate. Residents' perception of the adequacy of PCC practice was reclassified as adequate or inadequate, using a 50 mm cut-off point on VAS. Pearson's Chi-square was used to compare federal and state residents' responses and to assess for any significance between northern and southern geopolitical zones. A 2-sided *p* value < 0.05 was considered significant. The intraclass correlation coefficient was calculated for equipment and service adequacy. Specified challenges and recommendations were subjected to thematic analysis and presented in text boxes.

### 2.6. Ethical Consideration

Approval was obtained from the Research Ethics Committee (REC) of College of Medical Sciences, University of Benin (REC Approval no.: CMS/REC/2019/050). Permission was sought from the Local Organizing Committee of the NPMCN Intensive Course. Informed consent was obtained from every participant. Specific names and addresses of respondents' institutions were not required to ensure confidentiality.

## 3. Results

### 3.1. Baseline Characteristics of Participants and Their Institutions

A total of 143 paediatric trainees took part in the study, 37.1% of them were male, and 62.9% were female. Their mean age was 34.6 + 3.2 years. One hundred and two (72.9%) were registrars, 35 (25.0%) were senior registrars, and 3 (2.1%) were other cadres of trainees. They were mainly from federal tertiary institutions (86.7%). All parts of the country were represented with the south-south zone having the highest number of participants 44 (31.2%) and the north-eastern zone the least 10 (7.1%) (supplementary file Figure 1).

The commonest ICU facility available in the participants' institutions was general/mixed ICU (44.3%), followed by adult ICU (37.9%) and distantly by PICU (12.1%). The relative distribution of respondents with access to ICU facilities among the six geopolitical zones is shown in supplementary Table 1, while the distribution of those with access to PICU is shown in supplementary Figure 2. Less than half (45.5%) of the paediatric trainees had certification related to paediatric critical care and only 8 (11.9%) of them have done paediatric advanced life support (PALS) course. Further details of their baseline characteristics of the participants are as shown in Table 1.

### 3.2. Critical Care Needs in Participants' Self-Reported Clinical Practice

**Figure 1:**
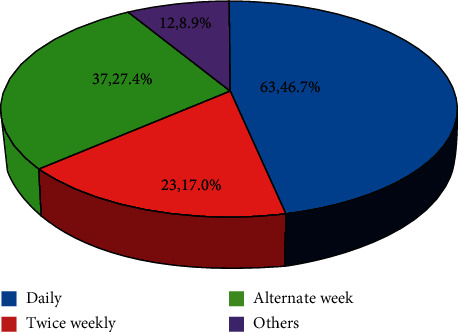
Frequency of treatment of critical illnesses in participants' clinical practice.

Less than a half (46.7%) of the trainees attend to critically ill children in their clinical practice daily, while 17.0% of the participants treat such children twice weekly. The rest of them encountered critical illness less frequently in their practice.  Figure 1 shows the frequencies of treatment of critical illness by the participants.

Also, most of them (83.8%) have recently managed children with severe respiratory distress; 29.8% of the respondents who managed such children reported that the children were admitted into ICU, while 74.6% of them said such patients eventually survived. Also, about a half of the participants have managed children in other Paediatric Assessment Triangle (PAT) categories with only 3 out of every 10 respondents stating that such children were able to assess ICU care. The commonest reasons for failed ICU admission were a “lack of appropriate equipment” and “financial constraint” (supplementary Table 2). Concerning the overall outcome, only 4 out of every 10 respondents stated that children with respiratory failure, decompensated shock, or cardiopulmonary failure survived till hospital discharge. The complete details on the types and outcome of the critical illnesses managed by the participants in their respective units in the preceding 3 weeks are shown in Table 2, using PAT classification.

Table 3 shows participants' level of agreement with the description of the nature and severity of critical illnesses seen in their clinical practice in the preceding 3 weeks. One hundred and nineteen participants (83.2%) adequately agreed with the descriptive statements on the nature and prognosis of the critical illnesses seen in their practice. Although the illnesses were infection-related and potentially reversible with critical care, late presentation to health facilities was common among the patients (3.51 ± 0.85; Table 3).

### 3.3. Distribution of Paediatric Intensive Care Resources in Various Zones of Nigeria

Limited functional PCC resources were available at the participants' institutions. The most widely available/functional resources were sphygmomanometers (79.4%), Ambu bags (25.8%), laryngoscopes (70.9%), and pulse oximeters (75.9%). The least available resources were capnographs (4.3%), mechanical ventilators (11.3%), syringe drivers (20.6%), and multiparameter monitors (31.9%). Sphygmomanometers, CPAP devices, multiparameter monitors, and pulse oximeters were relatively less available in the northern zones compared to the southern zones (*p* < 0.05). The complete zonal distribution of resources is shown in Table 4. The availability and functionality of resources in the PICUs are shown in supplementary file Table 3.

Altogether, human resources that were available in their training institutions include paediatric intensivists (23.6%) paediatricians (82.1%), ICU nurses (63.4%), ICU technicians (26.8%), and other cadres of ICU workers (4.9%). Forty-two trainees (29.4%) expressed a desire to specialize in paediatric intensive care. Paediatricians, ICU nurses, and ICU technicians were fewer in the northern zones than the southern zones, but there was no paediatric intensivist in the south-east zone (*p* < 0.05*;*Table 5).

### 3.4. Perceived Adequacy of PCC Resources, Challenges, and Recommendations

**Figure 2:**
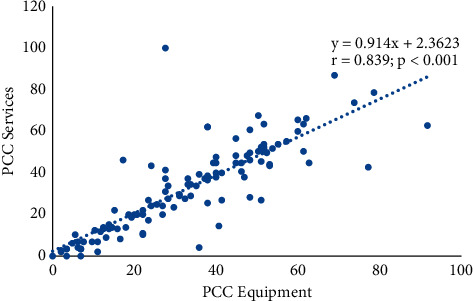
Relationship between perceived adequacy of PCC equipment and PCC services.

The mean VAS scores of the participants on 100 mm uncalibrated scales on the perceived adequacy of PCC equipment and PCC services in Nigeria were low, 32.7 ± 2.6 and 30.9 ± 2.8, respectively. There is a strong positive correlation between their perceived adequacy of equipment and services (*r* *=* 0.839; *p* < 0.001*;*Figure 2). Also, there is an excellent intraclass correlation coefficient (ICC) between the two measurements, ICC *=* 0.91 (95% CI: 0.87–0.94). Perceived challenges to PCC and recommendations by participants are listed as follows.

#### 3.4.1. Perceived Challenges

  Inadequacy or lack of equipment
  Poor staffing/no trained personnel
  Lack of regular training
  Lack of PICU bed space
  Financial constraint
  Unavailability of materials/drugs
  Late presentation of patients

#### 3.4.2. Recommendations by the Participants

  Establish paediatric ICU
  Make PICU equipment available
  Public-private partnership/funding
  Adequate training and retraining
  Employ more staff
  Subsidized health care services
  Improve power supply

## 4. Discussion

This study shows the unmet need for paediatric critical care in Nigeria with PICUs being only available to 12.1% of the respondents, similar to prior reports of limited critical care resources in subregions by other researchers [14–16]. There is a high burden of treatable severe childhood illnesses in the setting, encountered daily by nearly a half of the participants, but the majority of such children die due to the lack of optimal critical care, consistent with the single-centre report by Akindolire and Tongo [3] which reported that about a quarter of patients in their series die within 48 hrs of presentation in the emergency room (ER). Likewise, Nguefack et al. [12] in Cameroon found that death occurred early on admission especially among malnourished children. The aforementioned studies reported that most of the illnesses were infection-related and potentially reversible but the patients presented late to the referral centre, requiring intensive care services for survival [3, 12]. Factors promoting the delayed presentation of acutely ill children to health facilities in the setting are protean [11, 17]. Although they are being addressed at various levels, late presentation to the emergency room is still widely prevalent [10, 11]. Hence, there is a need to boost PCC service delivery in the country.

Moreover, basic monitoring equipment like sphygmomanometers and pulse oximeters are inadequate in over one-fifth of the training institutions, reflecting suboptimal care of acutely ill children and subsequently high paediatric mortality rates in the country [18, 19]. In this survey, ultramodern equipment like ventilators, capnographs, and multiparameter monitors were rarely available at the tertiary centres, consistent with earlier reports of major disparity between resource-limited and resource-rich centres by Muttalib et al. [4] and Abdelatif et al. [9] Similarly, Touray et al. [6] in Gambia and Siaw-Frimpong et al. [7] in Ghana reported less than 1 ICU bed/100,000 population in their countries. This highlights the need to improve health care financing and prioritize investment in critical care services in the region [20, 21]. Also, restructuring of the health system to include designated centres of excellence in PCC in various zones of the country may improve the availability of PCC resources nationwide in the public sector. This study found an intraclass correlation coefficient (ICC) of 0.91 between PCC equipment and services which is indicative of excellent reliability based on Koo and Li's guideline [22]. This confirms that the provision of the equipment is pertinent to enhancing PCC services in the setting.

Nonetheless, PCC human resources were not sufficiently available at the training institutions, perhaps partly due to the limited training opportunities in PCC in the setting as well as suboptimal healthcare financing. Brotherton et al. [23] in Kenya reported the creation of an Emergency and Critical Care Clinical Officer (ECCCO) higher diploma program since 2015 but there are currently no accredited degree or fellowship programs in PCC in Nigeria. Also, critical care-related courses like PALS are often expensive; only 11.9% of our participants had current certification in PALS. This highlights the need for collaboration with other postgraduate colleges for PCC training [23–25]. Several international training programs such as the Paediatric Emergency and Critical Care (PECC) Kenya fellowship training program, the African Paediatric Fellowships Program (APFP) in South Africa, and the Commonwealth Medical Fellowships program in the United Kingdom are available to build critical care capacity in resource-limited settings [26–28]. There should also be regular in-house training for all cadres of health workers as well as sponsored capacity-building workshops to enhance their PCC proficiency.

The main themes of perceived challenges to PCC in this national survey focus on poor staffing, lack of equipment, late presentation, and financial constraints, comparable to prior reports from resource-limited settings [9, 14, 16]. There is a need to upgrade service delivery at an affordable rate to the populace, considering the potentially catastrophic effects of out-of-pocket expenditures on families [29]. Recommendations stated in this survey to strengthen PCC comprise subsidizing healthcare services, possibly through the National Health Insurance Scheme (NHIS) and provision of more PICU bed spaces and relevant paediatric equipment in the country. These can be attained through viable NHIS and public-private partnership funding [21, 30].

This national survey has several strengths; its 100% response rate captures the views of all paediatric residents, attending the update course from all geopolitical zones of the country. The 3-week recall interval used in this study was short, ensuring the accurate provision of information. Also, many of the respondents were keenly aware of the dire need for PCC and the dearth of relevant resources in their institutions because they had spent 2 or more years in residency programs at their centres. Nonetheless, using the CROSS checklist [31], the limitations of this survey include the possibility of a recall bias and imprecision as well as a small sample size that can limit the generalizability of the findings. This sample size was estimated as 17.7% of paediatric trainees nationwide [32]. Future studies may focus on the on-site survey and key informant interviews at the training institutions to ascertain trainers' viewpoints. Also, a detailed assessment of critical care nursing manpower in the country is a desirable line of future study to strengthen PCC in the setting.

### 4.1. Conclusions

There is an unmet need for PCC practice in Nigerian tertiary hospitals with a resultant low survival rate of critically ill children in the setting. The scarcity of human resources can be ameliorated by incorporating relevant aspects of the PCC curriculum into the clinical training of health professionals as well as investing substantially into PCC physical resources.

## Figures and Tables

**Table 1:** Baseline characteristics of the study participants (*N* = 143).

Characteristics	Frequency, *n*	Percentage^*∗*^
Gender		
Male	53	37.1
Female	90	62.9

Status		
Registrar	102	72.9
Senior registrar	35	25.0
Others	3	2.1

Residency duration (years)		
< 2	80	56.7
2–4	29	20.6
˃4–6	32	22.7

Current institution		
Federal	124	86.7
State	16	11.2
Private/mission	3	2.1

Designation of institution		
University teaching hospital	88	62.0
Federal medical centre	46	32.4
Others	8	5.6

Location of institution		
North-central	21	14.9
North-east	10	7.1
North-west	21	14.9
South-south	44	31.2
South-west	26	18.4
South-east	19	13.5

ICU facility available		
PICU	17	12.1
Adult ICU	53	37.9
General/mixed ICU	62	44.3
None	8	5.7

Certification		
No	66	54.5
Yes	55	45.5

Type of certification		
BLS	53	79.1
PALS	8	11.9
Others	6	9.0

^*∗*^Percentages are denominated on a total number of respondents per variable (*N* ≤ 143).

**Table 2:** Types and outcomes of critical illnesses managed by the participants in the preceding 3 weeks (*N* = 136).

Critical illnesses	Managed, *n* (%)	Admitted ICU, *n* (%)^*∗*^	Total survived, *n* (%)^*∗*^
Severe respiratory distress	114 (83.8)	34 (29.8)	85 (74.6)
Respiratory failure	57 (41.9)	27 (47.4)	17 (29.8)
Compensated shock	75 (55.1)	11 (14.7)	44 (58.7)
Decompensated shock	49 (36.0)	13 (26.5)	17 (34.7)
CNS/metabolic dysfunction	71 (52.2)	16 (22.5)	25 (35.2)
Cardiopulmonary failure	70 (51.5)	14 (20.0)	20 (28.6)

^*∗*^Percentages are denominated on the number of respondents who managed the particular illness (*n* ≤ 114).

**Table 3:** Participant's agreement with description of critical illnesses seen in the preceding 3 weeks.

Description of critical illnesses	Agreement levels		
	Mean scores	SD	Interpretation
They are mainly infectious	2.92	1.06	Agree
They are potentially reversible disorders	3.15	0.64	Agree
Late presentation is common	3.51	0.85	Agree
They are metabolic disorders	2.36	0.79	Disagree
They are mainly congenital/genetic disorders	2.06	0.85	Disagree
Likely to survive with intensive care services	3.19	0.79	Agree

Reliability rating: Cronbach's alpha = 0.68.

**Table 4:** Distribution of paediatric intensive care resources in various zones of Nigeria^*∗*^.

Paediatric intensive care resources, *N*	North-central *n* (%)	North-east *n* (%)	North-west *n* (%)	South-south *n* (%)	South-west *n* (%)	South-east *n* (%)
Sphygmomanometer (paed cuffs), 123	22 (17.9)	9 (7.3)	18 (14.6)	35 (28.5)	22 (17.9)	17 (13.8)
Inotropic drugs, 100	17 (17.0)	9 (9.0)	11 (11.0)	32 (32.0)	17 (17.0)	14 (14.0)
Blood components, 71	9 (12.7)	4 (5.6)	16 (22.5)	18 (25.4)	17 (23.9)	7 (9.9)
CPAP devices, 61	6 (9.8)	4 (6.6)	12 (19.7)	17 (27.9)	13 (21.3)	9 (14.8)
Bubble CPAP, 90	15 (16.7)	9 (10.0)	15 (16.7)	25 (27.8)	13 (14.4)	13 (14.4)
Ambu bags/masks, 118	20 (16.9)	10 (8.5)	17 (14.4)	35 (29.7)	20 (16.9)	16 (13.6)
Laryngoscope/ET-tubes, 108	19 (17.6)	8 (7.4)	13 (12.0)	34 (31.5)	19 (17.6)	15 (13.9)
Oropharyngeal airways, 98	12 (12.2)	9 (9.2)	13 (13.3)	31 (31.6)	20 (20.4)	13 (13.3)
Multiparameter monitors, 54	4 (7.4)	4 (7.4)	12 (22.2)	20 (37.0)	9 (16.7)	5 (9.3)
Syringe drivers, 34	6 (17.6)	5 (14.7)	5 (14.7)	6 (17.6)	6 (17.6)	6 (17.6)
Mechanical ventilators, 43	9 (20.9)	4 (9.3)	8 (18.6)	8 (18.6)	9 (20.9)	5 (11.6)
AED/defibrillators, 21	3 (14.3)	0 (0.0)	8 (38.1)	2 (9.5)	6 (28.6)	2 (9.5)
ECG machine, 53	7 (13.2)	5 (9.4)	11 (20.8)	12 (22.6)	10 (18.9)	8 (15.1)
Capnograph (portable), 5	2 (40.0)	0 (0.0)	2 (40.0)	1 (20.0)	0 (0.0)	0 (0.0)
Pulse oximeters, 107	16 (15.0)	9 (8.4)	17 (15.9)	33 (30.8)	16 (15.0)	16 (15.0)

^*∗*^Percentages are denominated on the total number of respondents per variabl*e* (*N* *≤* 143); CPAP = continuous positive airway pressure.

**Table 5:** Distribution of intensive care human resources in various zones of Nigeria^*∗*^.

Human resources	North-central	North-east	North-west	South-south	South-west	South-east
Paediatric intensivists	9 (31.0)	2 (6.9)	6 (20.7)	8 (27.6)	4 (13.8)	0 (0.0)
Paediatricians^#^	15 (14.9)	7 (6.9)	13 (12.9)	30 (29.7)	23 (22.8)	13 (12.9)
ICU nurses	12 (15.4)	4 (5.1)	11 (14.1)	20 (25.6)	18 (23.1)	13 (16.7)
ICU technicians	5 (15.2)	2 (6.1)	1 (3.0)	8 (24.2)	10 (30.3)	7 (21.2)
Others	2 (33.3)	0 (0.0)	1 (16.7)	1 (16.7)	0 (0.0)	2 (33.3)

^*∗*^Percentages are denominated on the total number of respondents per variable (*N* ≤ 143). ^#^Paediatricians with interest in intensive care.

## Data Availability

The completed questionnaires and excel spreadsheet of the research reported in this article are available from the authors on request.

## Supplementary Materials

Supplementary Materials Figure 1: relative distribution of respondents among the six geopolitical zones of Nigeria, one state representing each zone (specific addresses of the training institutions not included) (powered by Bing© GeoNames, Microsoft, Tom Tom). Figure 2: relative distribution of respondents with access to PICU among the six geopolitical zones, one state representing each zone (specific addresses of training institutions not included) (powered by Bing© GeoNames, Microsoft, Tom Tom). Table 1: distribution of respondents with access to intensive care facilities by geopolitical zones. Table 2: reasons for not admitting critically ill children into ICU indicated by the respondents (*N* = 136). Table 3: availability and functionality of PICU resources indicated by the respondents (*N* = 17). .
