# Supplementary material for: Evaluation of Paediatric Critical Care Needs and Practice in Nigeria: Paediatric Residents' Perspective
Source: Crit Care Res Pract. 2021 Aug 31;2021:2000140. doi: 10.1155/2021/2000140 (PMC8426102; doi:10.1155/2021/2000140)
Supplement: Supplementary Materials — Figure 1: relative distribution of respondents among the six geopolitical zones of Nigeria, one state representing each zone (specific addresses of the training institutions not included) (powered by Bing© GeoNames, Microsoft, Tom Tom). Figure 2: relative distribution of respondents with access to PICU among the six geopolitical zones, one state representing each zone (specific addresses of training institutions not included) (powered by Bing© GeoNames, Microsoft, Tom Tom). Table 1: distribution of respondents with access to intensive care facilities by geopolitical zones. Table 2: reasons for not admitting critically ill children into ICU indicated by the respondents (N = 136). Table 3: availability and functionality of PICU resources indicated by the respondents (N = 17). . [file 2000140.f1.zip › 2000140.f1/Supplementary Table 1.docx]

**Supplementary Table 1**: **Distribution of respondents with access to intensive care facilities by geopolitical zones**

| **ICU Facilities** | **North Central**  **n (%)** | **North East**  **n (%)** | **North West**  **n (%)** | **South-South**  **n(%)** | **South East**  **n(%)** | **South West**  **n(%)** |
| --- | --- | --- | --- | --- | --- | --- |
| **PICU** | 1(5.9) | 0 (0.0) | 3(16.7) | 6(17.1) | 3(17.6) | 3 (13.6) |
| **Adult ICU** | 9(52.9) | 3(33.3) | 10(55.6) | 18(51.4) | 6(35.3) | 6(27.3) |
| **Mixed ICU** | 10(58.8) | 6(66.7) | 6(33.3) | 15(42.9) | 10(58.8) | 14(63.6) |
