# Supplementary material for: Evaluation of Paediatric Critical Care Needs and Practice in Nigeria: Paediatric Residents' Perspective
Source: Crit Care Res Pract. 2021 Aug 31;2021:2000140. doi: 10.1155/2021/2000140 (PMC8426102; doi:10.1155/2021/2000140)
Supplement: Supplementary Materials — Figure 1: relative distribution of respondents among the six geopolitical zones of Nigeria, one state representing each zone (specific addresses of the training institutions not included) (powered by Bing© GeoNames, Microsoft, Tom Tom). Figure 2: relative distribution of respondents with access to PICU among the six geopolitical zones, one state representing each zone (specific addresses of training institutions not included) (powered by Bing© GeoNames, Microsoft, Tom Tom). Table 1: distribution of respondents with access to intensive care facilities by geopolitical zones. Table 2: reasons for not admitting critically ill children into ICU indicated by the respondents (N = 136). Table 3: availability and functionality of PICU resources indicated by the respondents (N = 17). . [file 2000140.f1.zip › 2000140.f1/Supplementary Table 3.docx]

**Supplementary Table 3: Availability and functionality of PICU resources indicated by the respondents (N = 17)**

| **PICU Resources** | **Available** | | **Functional^*^** | |
| --- | --- | --- | --- | --- |
|  | **n** | **%** | **n** | **%** |
| Sphygmomanometer [paed cuffs] | 12 | 70.6 | 13 | 76.5 |
| Inotropic drugs | 12 | 70.6 | 10 | 58.8 |
| Blood components | 10 | 58.8 | 8 | 47.1 |
| CPAP Devices | 11 | 64.7 | 11 | 64.7 |
| Bubble CPAP | 11 | 64.7 | 12 | 70.6 |
| Ambubags/ Masks | 14 | 82.4 | 17 | 100.0 |
| Laryngoscope/ ET-tubes | 13 | 76.5 | 15 | 88.2 |
| Oropharyngeal airways | 12 | 70.6 | 11 | 64.7 |
| Multi-parameter monitors | 8 | 47.1 | 7 | 41.2 |
| Syringe drivers | 4 | 23.5 | 5 | 29.4 |
| Mechanical Ventilators | 3 | 17.6 | 0 | 0.0 |
| AED / defibrillators | 2 | 11.8 | 0 | 0.0 |
| ECG machine | 4 | 23.5 | 3 | 17.6 |
| Capnograph (portable) | 2 | 11.8 | 0 | 0.0 |
| Pulse oximeters | 12 | 70.6 | 12 | 70.6 |

**Some respondents only indicated functional resources in their PICUs*
