# Supplementary figures and images for: Evaluation of Paediatric Critical Care Needs and Practice in Nigeria: Paediatric Residents' Perspective
Source: Crit Care Res Pract. 2021 Aug 31;2021:2000140. doi: 10.1155/2021/2000140 (PMC8426102; doi:10.1155/2021/2000140)

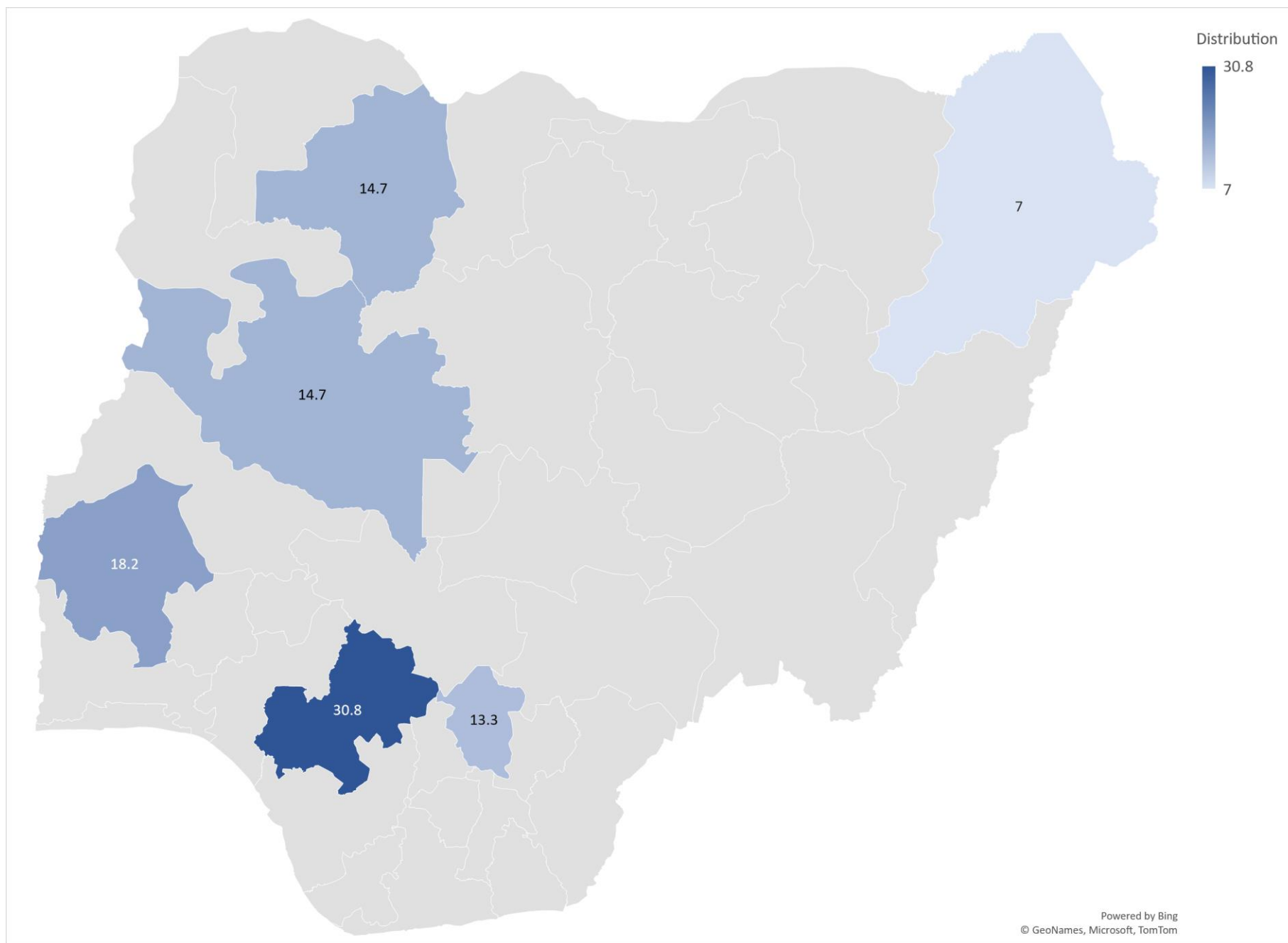

Supplement: Supplementary Materials — Figure 1: relative distribution of respondents among the six geopolitical zones of Nigeria, one state representing each zone (specific addresses of the training institutions not included) (powered by Bing© GeoNames, Microsoft, Tom Tom). Figure 2: relative distribution of respondents with access to PICU among the six geopolitical zones, one state representing each zone (specific addresses of training institutions not included) (powered by Bing© GeoNames, Microsoft, Tom Tom). Table 1: distribution of respondents with access to intensive care facilities by geopolitical zones. Table 2: reasons for not admitting critically ill children into ICU indicated by the respondents (N = 136). Table 3: availability and functionality of PICU resources indicated by the respondents (N = 17). . [file 2000140.f1.zip › 2000140.f1/Supplementary Figure 1 Participants Distribution Map (1).pdf]

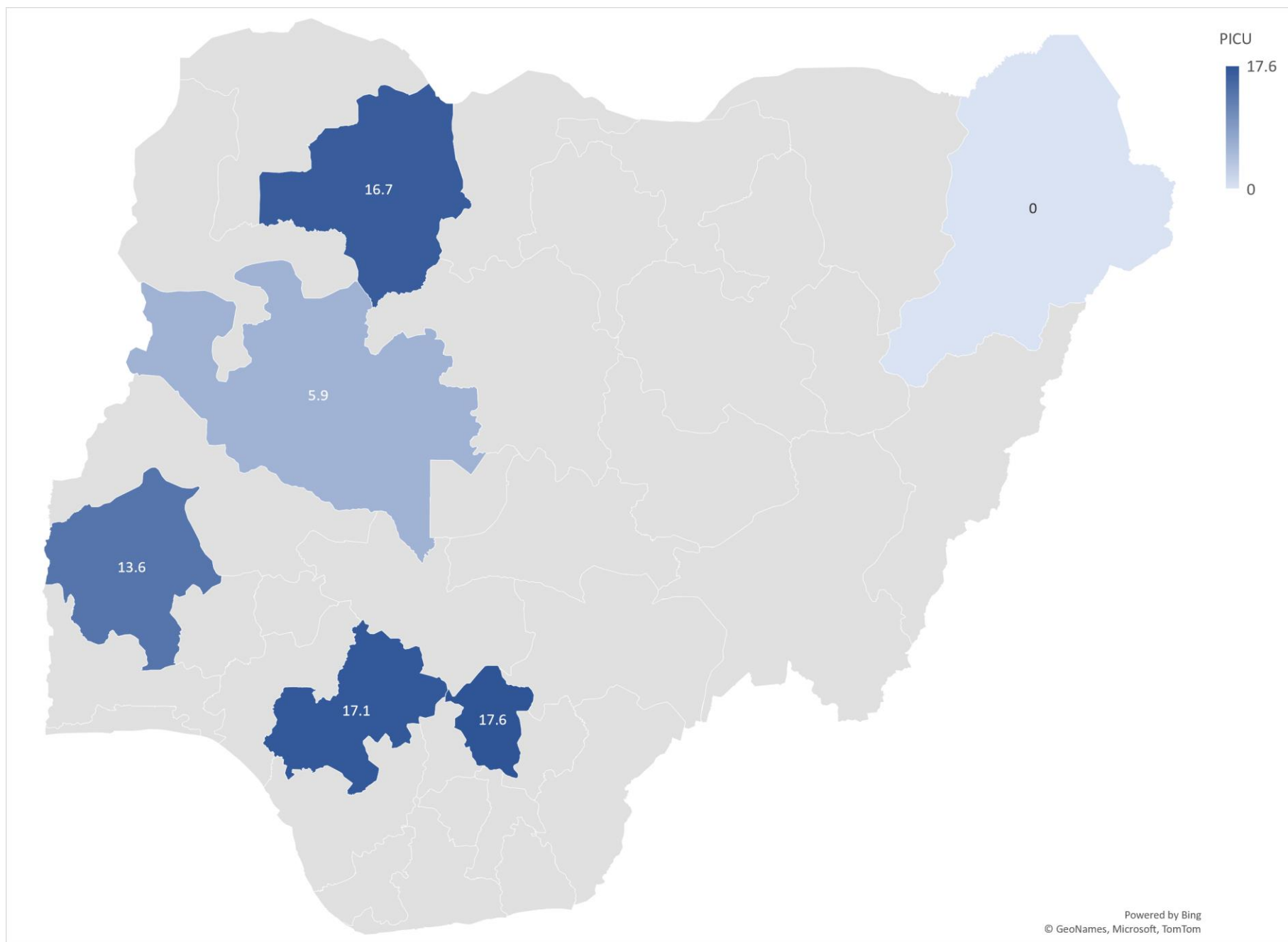

Supplement: Supplementary Materials — Figure 1: relative distribution of respondents among the six geopolitical zones of Nigeria, one state representing each zone (specific addresses of the training institutions not included) (powered by Bing© GeoNames, Microsoft, Tom Tom). Figure 2: relative distribution of respondents with access to PICU among the six geopolitical zones, one state representing each zone (specific addresses of training institutions not included) (powered by Bing© GeoNames, Microsoft, Tom Tom). Table 1: distribution of respondents with access to intensive care facilities by geopolitical zones. Table 2: reasons for not admitting critically ill children into ICU indicated by the respondents (N = 136). Table 3: availability and functionality of PICU resources indicated by the respondents (N = 17). . [file 2000140.f1.zip › 2000140.f1/Supplementary Figure 2 PICU distribution Map (1).pdf]
